# The associations among childhood trauma, loneliness, mental health symptoms, and indicators of social exclusion in adulthood: A UK Biobank study

**DOI:** 10.1002/brb3.2959

**Published:** 2023-03-15

**Authors:** Sarah F. Allen, Simon Gilbody, Karl Atkin, Christina M. van der Feltz‐Cornelis

**Affiliations:** ^1^ Department of Psychology Northumbria University Newcastle upon Tyne UK; ^2^ Department of Health Sciences University of York York UK; ^3^ Hull York Medical School University of York York UK; ^4^ York Biomedical Research Institute University of York York UK

**Keywords:** childhood trauma, loneliness, mental health, social exclusion, UK Biobank

## Abstract

**Aims:**

Childhood trauma has been associated with adult psychosocial outcomes linked to social exclusion. However, the strength of these associations in the general population is unknown. The emergence of the UK Biobank, with rich phenotypic characterization of the adult population, affords the exploration of the childhood determinants of adult psychopathology with greater statistical power. The current study aims to explore (1) the associations between childhood trauma and social exclusion in adulthood and (2) the role that self‐reported loneliness and symptoms of distress play in the associations.

**Methods:**

This study was an analysis of 87,545 participants (mean [± SD] age = 55.68 [7.78], 55.0% female, 97.4% White) enrolled in the UK Biobank. Childhood trauma was determined by the five‐item Childhood Trauma Screener. Current loneliness and symptoms of anxiety (Generalized Anxiety Disorder Scale‐7) and depression (Patient Health Questionnaire‐9) were also entered in analyses. Outcomes were “limited social participation,” “area deprivation,” “individual deprivation,” and “social exclusion” from a previously determined dimensional measure of social exclusion in the UK Biobank.

**Results:**

Hierarchical multiple regression models indicated small associations between childhood trauma and social exclusion outcomes, explaining between 1.5% and 5.0% of the variance. Associations weakened but remained significant when loneliness, anxiety, and depression were entered in the models; however, anxiety symptoms demonstrated a negative association with “individual deprivation” and “social exclusion” in the final models. Depression was most strongly associated with “individual deprivation,” “area deprivation,” and “social exclusion” followed by childhood trauma. Loneliness was most strongly associated with “limited social participation.”

**Conclusions:**

Experiences of childhood trauma can increase the propensity for adulthood social exclusion. Loneliness and symptoms of depression attenuate but do not eliminate these associations. Anxiety symptoms have a potentially protective effect on the development of “individual deprivation.” Findings add to the growing body of literature advocating for trauma‐informed approaches in a variety of settings to help ameliorate the effects of childhood trauma on adult psychosocial outcomes. Further research, however, is required.

## Graphical abstract

An analysis of UK Biobank data finds associations between childhood trauma and increased indicators of social exclusion. These associations are attenuated by but not eliminated by loneliness and depression. Anxiety may play a minor protective role in the development of employment and education related aspects of social exclusion.

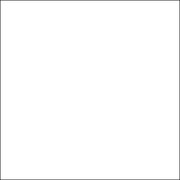

## Significant Outcomes

- More occurrences of trauma and neglect in childhood are associated with an increase in indicators of social exclusion in adulthood.
- Loneliness and symptoms of depression exacerbate the associations between childhood trauma and the propensity for the development of social exclusion.
- Anxiety symptoms may play a minor protective role against individual‐level deprivation with respect to education and employment.

## Limitations

- The analyses in the current study are exploratory and retrospective, and therefore direct causal pathways cannot be inferred.
- Examination of familial factors (e.g., composition) and socioeconomic status in childhood would have been good to include. However, there is an absence of data in the UK Biobank relating to these factors.
- The UK Biobank is a large but not entirely representative sample of the U.K. general population.

## 1 INTRODUCTION

Converging evidence from neurobiology, psychiatry, and epidemiology has shown that the experience of childhood trauma such as physical and sexual abuse, emotional neglect, social fragmentation, and poverty leads to an increased risk of psychopathology (Anda et al., 2006; Chapman et al., 2004; Felitti et al., 1998; Lippard & Nemeroff, 2020; Mondelli & Dazzan, 2019; Nemeroff, 2016; Sachs‐Ericsson et al., 2005). It is estimated that around half of individuals in the mental healthcare system have experienced physical abuse, and more than one‐third sexual abuse, as children (Mauritz et al., 2013) further exemplifying this link. A nationally representative survey of residents in the United Kingdom aged 18–69 (Bellis et al., 2014) found 6.3% of participants reported experiences of childhood sexual abuse; 14.8% of physical abuse and 18.2% of verbal abuse indicate it is a widespread issue.

Individuals exposed to adverse experiences in childhood have a higher likelihood of developing psychiatric conditions (Chapman et al., 2004), and have poor physical health outcomes (Felitti et al., 1998) as adults. For example, survivors of abuse in childhood show higher adult rates of persistent physical symptoms (Lamahewa et al., 2019), chronic illness (Mock & Arai, 2011), somatic comorbidity (Van der Feltz‐Cornelis et al., 2019), and chronic pain in later life (Davis et al., 2005). Taken together, this research exemplifies the need to consider the influence of trauma exposure as a key mental and physical health priority.

Experiencing trauma in childhood can hamper socioemotional and interpersonal development skills in adulthood via dysregulation of emotional responses (Poole et al., 2018). This can engender negative beliefs and attitudes toward other people (Kendall‐Tackett, 2002) and increase participation in risky activities, both of which can result in societal integration problems for those individuals. Further, research has demonstrated that those who have experienced trauma in childhood are at increased risk of homelessness and engaging in antisocial behavior (Gibson & Hartshorne, 1996; Gilbert et al., 2009; Wilson & Widom, 2009).

Adult survivors of childhood adversity are less likely to seek support from their peers and may choose to avoid forming or maintaining relationships altogether, putting themselves at greater risk of social isolation (Gibson & Hartshorne, 1996). Difficulties integrating into society can be exacerbated by, and lead to, social isolation, loneliness, and mental health difficulties (Boardman, 2011). Often individuals with mental health problems may find themselves excluded from mainstream society due to stigma and lack of support, whereas elements of social exclusion such as poor social networks, unemployment, lack of access to services, and poverty can exacerbate existing psychopathologies (Boardman, 2011). Multiple studies have also reported a higher prevalence of childhood adversity in populations in which social exclusion, particularly economic deprivation, is high (Bellis et al., 2014; Ramaesh et al., 2015).

From a life course perspective, a cumulative model can be proposed. Early life stress stemming from the experience of childhood abuse and neglect can lead to adversity in later life (Gilbert et al., 2009), but also make individuals more vulnerable to later‐life distress via psychophysiological dysregulation (Dich et al., 2015). This can result in individuals who have experienced childhood trauma being more likely to report increased distress and mental health symptoms in adulthood, which in turn can influence educational and occupational outcomes, as well as the ability to integrate fully in society (Seery et al., 2010).

The term “social exclusion” is so rarely clearly defined, but essentially conceptualizes the concept of “disadvantage” and is traditionally viewed in terms of material deprivation, characterized by poverty and unemployment (Levitas, 2006). There has, however, been a recent shift toward the view that social exclusion is a relative concept that focuses on the idea that participation is key; and lack of participation is due to constraints rather than choices (Boardman, 2011). Poverty and unemployment do not necessarily lead to exclusion from society despite both having significant effects on some but not all aspects of social participation (Levitas, 2006). The Economic and Social Research Council more recently defined social exclusion as “the processes by which individuals and their communities become polarized, socially differentiated, and unequal.” There have been many debates over the indicators of social exclusion relating to both issues of definition and availability of appropriate data (Levitas, 2006). Taken together, there is no clear consensus on the assessment of social exclusion, other than that its measurement is complex and multidimensional. Nevertheless, recent recommendations encourage the consideration of social status, finances, community roles, disability, and access to services (Van Bergen et al., 2017).

Considering these recommendations, a social exclusion construct was recently developed from UK Biobank data to enable large‐scale population‐level research into the causes and consequences of the concept. Although admittedly the data available were unable to provide all factors theorized to underpin social exclusion, the developed construct was found to comprise three factors: (1) “limited social participation” based on social networks and community integration; (2) “individual deprivation” based on education level, employment status, and income; and (3) “area deprivation” based on deprivation scores in current U.K. area of residence (Allen et al., 2020). This measure will be implemented in the current study to assess the links between childhood trauma and social exclusion within the UK Biobank data.

The UK Biobank is a large‐scale general population sample comprising biological data and health assessments of over 500,000 volunteers in the United Kingdom (Sudlow et al., 2015). While conceived to explore the genetic basis of health, it also provides rich phenotypic data and characterization of childhood exposures and adult mental health psychosocial variables pertinent to the aims of this study. Previous UK Biobank studies have identified relationships between childhood trauma and various psychological and social factors (e.g., Bauermeister & Gallacher, 2018; Coleman et al., 2020); yet no studies from this sample have investigated the effects of recalled childhood adversity on later‐life indicators of social exclusion. The current study therefore aims to use UK Biobank data to explore (1) the associations between childhood trauma and social exclusion in adulthood; and (2) the role that self‐reported loneliness and symptoms of distress play in these associations.

Given previous findings indicating that childhood trauma is linked to an increased propensity for social isolation and issues integrating into society, it is hypothesized that higher levels of childhood trauma will be associated with increased social exclusion, and loneliness, anxiety, and depression will contribute to these associations.

## 2 METHOD

### 2.1 Study design

This was a cross‐sectional population‐based study of participants enrolled in the UK Biobank including a retrospective assessment of childhood trauma.

### 2.2 Study setting and sample

The UK Biobank recruited 502,655 participants by postal invitation between March 2006 and October 2010. Participants attended one of 22 assessment centers across England, Scotland, and Wales, where they completed touchscreen and nurse‐led questionnaires, had physical measurements taken, and provided biological samples. All individuals registered with the National Health Service (NHS) aged 40−69 years living within 25 miles radius of a study assessment center were invited to participate. Participants for the current study were those who completed the baseline assessment questionnaires between 2006 and 2010, plus the online mental health follow‐up between 2016 and 2019 (Davis et al., 2020). Participants who provided data on childhood trauma, mental health symptoms, and social exclusion were included in the analyses. As determined by the pertinent data available, the final sample size equaled *N* = 87,545.

### 2.3 Ethics statement

UK Biobank received ethical approval from the NHS National Research Ethics Service North‐West (11/NW/0382). Written informed consent was obtained from participants at all stages of data collection.

### 2.4 Variables

#### 2.4.1 Predictor variable

The validated five‐item Childhood Trauma Screener (CTS‐5; Grabe et al., 2012) was used for the retrospective assessment of adverse experiences prior to age 18. Questions covered sexual, emotional, and physical abuse and emotional and physical neglect. Responses ranged from 0 = *never* to 4 = *very often true*. Two positively worded questions were reverse scored, and the summation of responses gave a total score for analysis. Higher scores indicated higher levels of childhood trauma. Responses of “prefer not to answer” were treated as missing data.

The prevalence of childhood trauma was also calculated and reported in Table 1. A cutoff score of >1 on the abuse questions or >2 on the neglect questions indicated experience of “any trauma.”

**TABLE 1:** Description of the sample (*n* = 87,545)

Variable		*n*/mean [± SD])	Missing data (%)
Age at baseline assessment		55.68 [7.78]	0%
Sex	Female	48,114 (55%)	0%
	Male	39,431 (45%)	
Ethnic background	White	85,079 (97.4%)	0.2%
	Mixed	460 (0.5%)	
	Asian/Asian British	670 (0.8%)	
	Black/Black British	564 (0.6%)	
	Chinese	151 (0.2%)	
	Other	433 (0.5%)	
	Undeclared	188 (0.2%)	
CTS‐5—Childhood trauma (mean [± SD])		3.10 [1.92]	3.1%
Regularly felt loved as a child (% no)		6.1%	
Regularly hated by a family member (% yes)		2.9%	
Regularly had someone to take them to the doctor (% no)		2.9%	
Ever physically abused (% yes)		18.9%	
Ever sexually molested (% yes)		8.9%	
Any trauma (%)		29.0%	
Loneliness (% yes)		15.9%	
Limited social participation (mean [± SD])		6.81 [2.49]	2.1%
Individual deprivation (mean [± SD])		6.86 [2.77]	0.4%
Area deprivation (mean [± SD])		8.00 [3.77]	3.0%
Total social exclusion (mean [± SD])		21.66 [5.93]	5.1%
PHQ‐9‐Depression	(Mean [± SD])	2.77 [3.72]	1.6%
	% of n = Normal/mild (<10)	93.9%	
	% of n = Moderate/severe (10+)	6.1%	
GAD‐7‐Anxiety	(Mean [± SD])	2.30 [3.56]	1.0%
	% of n = Normal/mild (<10)	95.0%	
	% of n = Moderate/severe (10+)	95.0%	

#### 2.4.2 Outcome variables

The main outcome variables were the indicators of social exclusion as assessed by the dimensional construct outlined in a recent paper (Allen et al., 2020), which used UK Biobank data (*n* = 502,655) to calculate the following components of social exclusion: “limited social participation” (items relating to a lack of social support, limited leisure activities, and the inability to confide in others); “area deprivation” (a composite measure of IMD scores), and “individual deprivation” (household income, education level, and employment status). The data were treated in the same manner as the original paper and a score between 0 and 4 was calculated for each of the three dimensions. The separate scores were also then summed to provide an additional total social exclusion score of 0–12. Higher scores indicated higher levels of social exclusion indicators.

#### 2.4.3 Secondary predictor variables

Loneliness and symptoms of anxiety and depression were included as secondary predictor variables.

Loneliness was a categorical variable that was assessed by a single question “Do you feel lonely?” Response options were “yes” or “no” and participants were categorized as “not lonely” = 0 or “lonely” = 1.

Anxiety symptoms were assessed by the Generalized Anxiety Disorder Scale (GAD‐7; Spitzer et al., 2006), a validated seven‐item questionnaire that asks about the frequency of anxiety symptoms over the past 2 weeks from *not at all* (0) to *every day* (3). Total scores ranged between 0 and 21 with higher scores equal to more symptoms of anxiety.

Depressive symptoms were assessed by the validated nine‐item Patient Health Questionnaire (PHQ‐9; Kroenke & Spitzer, 2002), which asks about the frequency of depression symptoms over the past 2  weeks. Response options range from *not at all* (0) to *every day* (3). Total scores range between 0 and 27. Higher scores indicate more symptoms of depression.

Symptoms of anxiety and depression were kept as continuous data in analyses to assess their linear predictive value in line with the research question. However, utilizing the cutoff score of >10 on both measures, the prevalence of clinically significant anxiety and depression was calculated and is reported in Table 1.

### 2.5 Demographics

Data on age, biological sex, and ethnic origin were also collected to demonstrate the demographic characteristics of the sample.

### 2.6 Statistics

IBM SPSS version 26.0 was used to analyze the data. Missing data, while anticipated to have minimal influence in such a large data set, were treated using listwise deletion (see Table 1). While this method of missing data treatment can increase bias, this is already an existing limitation of the UK Biobank data; therefore, it is acknowledged that conclusions regarding representativeness cannot be drawn. Correlational analyses assessed the linear relationships among the predictors, outcomes, age, and sex. The distribution of scores on the CTS‐5, GAD7, and PHQ9 were positively skewed; therefore, Spearman's rho correlations were conducted. Point biserial correlations were conducted for relationships between the variables of loneliness and sex. Reference categories were “not lonely” and “female” (i.e., = 0) for analysis.

Four hierarchical multiple regression models using the enter method were conducted to assess the predictive value of childhood trauma for each social exclusion outcome. To assess the independent contribution of each predictor, childhood trauma was entered in Step 1, loneliness was added in Step 2, and anxiety and depression in Step 3.

## 3 RESULTS

Demographic data and descriptive statistics are reported in Table 1.

As shown in Table 2, small significant positive (*r* = .10–.30, *p*s < .001) relationships were observed between the occurrence of childhood trauma with each of the secondary predictors and all social exclusion outcomes. Negligible (*r* < .01) negative correlations were observed with age and sex; therefore, it was decided these variables would not be included in the regression analyses.

**TABLE 2:** Correlations between the dimensions of social exclusion with scores on the CTS‐5, PHQ‐9, and GAD‐7, and loneliness, age, and sex

	Childhood trauma	Loneliness	Limited social participation	Individual deprivation	Area deprivation	Social exclusion	PHQ9	GAD7	Age
Loneliness	.138^**^	–							
Limited social participation	.107^**^	.174^**^	–						
Individual deprivation	.167^**^	.152^**^	.051^**^	–					
Area deprivation	.136^**^	.098^*^	.090^**^	.298^**^	–				
Social exclusion	.203^**^	.201^**^	.473^**^	.641^**^	.801^**^	–			
PHQ9	.189^**^	.301^**^	.134^**^	.159^**^	.144^**^	.212^**^			
GAD7	.162^**^	.255^**^	.090^**^	.090^**^	.096^**^	.133^**^	.660^**^		
Age at baseline	–.023^**^	–.034^**^	–.050^**^	.366^**^	–.057^**^	.106^**^	–.119^**^	–.129^**^	
Sex	–.061^**^	–.044^**^	.118^**^	–.028^**^	.007	.040^**^	–.078^**^	–.101^**^	.061^**^

^*^ *p* < .05; ^**^ *p* < .01.

No evidence of multicollinearity between the predictor variables was observed (tolerance values were greater than .20 and Variance Inflation factors [VIFs] between 1 and 2). Table 3 presents the results of the regression analysis.

**TABLE 3:** Hierarchical regression analyses indicating associations between childhood trauma and each social exclusion outcome. Loneliness is added at Step 2, and anxiety and depression added at Step 3

	*B*	SE *b*	*β*	*t*	*p*	*R* ^2^ (Δ*R* ^2^)
Model 1: Limited social participation						
Childhood trauma	.123	.009	.124	14.292	<.001	.015^**^
Childhood trauma	.098	.009	.099	11.425	<.001	
Loneliness	1.010	.051	.170	19.671	<.001	.044 (.028)^**^
Childhood trauma	.078	.009	.079	8.967	<.001	
Loneliness	.829	.053	.140	15.514	<.001	
PHQ9	.063	.006	.120	9.900	<.001	
GAD7	–.010	.007	–.017	–1.468	.142	.054 (.010)^**^
Model 2: Individual deprivation						
Childhood trauma	.190	.009	.181	21.037	<.001	.033^**^
Childhood trauma	.170	.009	.162	18.813	<.001	
Loneliness	.791	.054	.126	14.563	<.001	.048 (.015)^**^
Childhood trauma	.145	.009	.138	15.796	<.001	
Loneliness	.558	.056	.088	9.884	<.001	
PHQ9	.090	.007	.161	13.293	<.001	
GAD7	–.023	.007	–.038	–3.214	.001	.065 (0.17)^**^
Model 3: Area deprivation						
Childhood trauma	.217	.013	.148	17.107	<.001	.022^**^
Childhood trauma	.201	.013	.137	15.707	<.001	
Loneliness	.652	.077	.074	8.478	<.001	.027 (.005)^**^
Childhood trauma	.169	.013	.115	12.985	<.001	
Loneliness	.357	.080	.040	4.465	<.001	
PHQ9	.102	.010	.130	10.651	<.001	
GAD7	–.015	.010	–.017	–1.407	.159	.040 (.012)^**^
Model 4: Social exclusion						
Childhood trauma	.529	.020	.224	26.346	<.001	.050^**^
Childhood trauma	.469	.020	.199	23.439	<.001	
Loneliness	2.453	.120	.173	20.416	<.001	.080 (.029)^**^
Childhood trauma	.391	.020	.166	19.450	<.001	
Loneliness	1.744	.124	.123	14.082	<.001	
PHQ9	.255	.015	.203	17.206	<.001	
GAD7	–.048	.016	–.035	–3.006	.003	.108 (.029)^**^

^*^ *p* < .05; ^**^ *p* < .01.

Model 1 was significant at Step 1 (*F*(1, 87,544) = 204.26, *p* < .05), indicating that the occurrence of childhood trauma explained 1.5% of the variance in “limited social participation.” The additions of loneliness at Step 2 (*p* < .05) and anxiety and depression at Step 3 (*p* < .05) significantly increased the amount of variance explained. The final model explained a total of 5.4% of the variance in “limited social participation.” The strongest association was with loneliness, followed by depressive symptoms (*β* = .120) and childhood trauma (*β* = .079). Anxiety was not a significant predictor in the final model.

Model 2 was significant at Step 1 (*F*(1, 87,544) = 442.56, *p* < .05), indicating that childhood trauma explained 3.3% of the variance in “individual deprivation.” The model was significant at Step 2 (*p* < .05) and Step 3 (*p* < .05). The final model explained 6.5% of the variance in individual deprivation with depression (*β* = .161) as the strongest predictor, followed by childhood trauma (*β* = .138) and loneliness (*β* = .088). Anxiety symptoms (*β* = –.038) were negatively associated with the outcome.

Model 3 was also significant at Step 1 (*F*(1, 87,544) = 292.65, *p* < .05), demonstrating that childhood trauma could explain 2.2% of the variance in “area deprivation.” The model was also significant at Step 2 (*p* < .05) and at Step 3 (*p* < .05). The final model explained 4.0% of the variance in “area deprivation.” Again, depression (*β* = .130) was the strongest predictor, closely followed by childhood trauma (*β* = .115) and finally loneliness (*β* = .048). Anxiety was not a significant predictor in the final model.

Model 4 was also significant at Step 1 (*F*(1, 87,544) = 694.09, *p* < .05), indicating that the occurrence of childhood trauma explained 5.0% of the variance in social exclusion. The model was again significant at Step 2 (*p* < .05) and at Step 3 (*p* < .05). The final model explained 10.8% of the variance in “Social exclusion” with depression (*β* = .203) again as the strongest predictor, followed by childhood trauma (*β* = .166) and loneliness (*β* = .088). Anxiety (*β* = –.035) was weakly negatively associated with the combined social exclusion outcome.

## 4 DISCUSSION

The current study aimed to explore the associations between childhood trauma and social exclusion in the UK Biobank. The prevalence of childhood trauma (at least one occurrence) in the sample was 29%, which, compared to percentages reported in previous studies in the United Kingdom (21.1%) (Bellis et al., 2014) and Australia (10.0%) (Draper et al., 2008), is clearly larger. However, this could be due to inconsistencies in the assessment and classification of childhood trauma across studies. As such, if we consider only instances of physical abuse (18.9%) and sexual molestation (8.9%) in the current study, the prevalence rates are closer to that of previous studies.

As expected, our results indicated that higher occurrences of childhood trauma were associated with increased indicators of social exclusion. This was demonstrated for each social exclusion dimension in isolation and combined. These findings support existing literature demonstrating relationships between early‐life adversity and social outcomes in later life (Gibson & Hartshorne, 1996; Gilbert et al., 2009; Shelton et al., 2009).

Self‐reported loneliness and heightened anxiety and depressive symptoms were also independently associated with both increased social exclusion and childhood trauma in the current analyses. The regression analyses demonstrated that while childhood trauma predicted all social exclusion outcomes, effects were attenuated, but not eliminated, by loneliness and depression, garnering support for existing causal evidence of the complex links among childhood trauma, psychosocial difficulties, and depression in adulthood (Bentall et al., 2014; Conroy et al., 2010; Felitti et al., 1998; Kendall‐Tackett, 2002; Radell et al., 2021; Springer et al., 2007).

In the final models, anxiety symptoms were negatively associated with individual deprivation and social exclusion, and the effects of anxiety diminished in the prediction of “limited social participation” and “area deprivation.” Given the composition of the “individual deprivation” dimension (i.e., employment, education, income), this may tentatively suggest that low levels of anxiety could have a positive impact on individuals’ pursuit of education and employment, potentially in line with the theory that the Yerkes and Dodson law (i.e., there is an inverted U‐shaped relationship between arousal and cognitive performance; Yerkes & Dodson, 1908) may apply to anxiety symptoms, particularly given that average levels of anxiety were low in the current sample (only 5% had a clinically significant GAD‐7 score of above 10). Alternatively, as the scores on the PHQ‐9 and GAD7 were highly correlated, it could also be suggested that the negative effect of anxiety could be due to collinearity between the measures.

Depressive symptoms were the strongest predictor of “individual deprivation,” “area deprivation,” and “social exclusion,” consequently agreeing with previous research demonstrating bidirectional relationships between mental illness and social exclusion (e.g., Boardman, 2011; Morgan et al., 2007). Childhood trauma was the second strongest predictor of these outcomes, and while the predictive value was small, and the effects were attenuated by loneliness and depression, the analyses suggested that every increase of 1 SD in scores on the CTS‐5 would lead to a rise of around 0.1–0.2 SDs in “individual deprivation,” “area deprivation,” and “social exclusion.” Sadly, cumulative trauma exposure and social exclusion are not uncommon issues in the United Kingdom (Stewart et al., 2023).

The finding that childhood trauma can predict an increase in “individual deprivation” is also interesting, and it supports evidence of poor educational achievement and motivation in individuals with a history of early‐life adversity (e.g., Crosby, 2015; Keller‐Dupree, 2013; Pereira et al., 2018). However, if anxiety plays a protective role against individual deprivation as the findings tentatively suggest. Under some circumstances, anxiety has been found to enhance aspects of cognition (Robinson et al., 2013), and longitudinal evidence has shown that the pursuit of education and stable employment is a central component of overcoming early life adversity (Werner, 2013). This finding is potentially suggestive of a contradictory moderation effect, similar to the healthy neuroticism hypothesis, that suggests that high neuroticism (which is usually associated with poorer physical and mental health outcomes) when combined with high conscientiousness can have positive effects (Friedman, 2000). Therefore, anxiety in the sense of vigilance from further adversity may very well prevent people from social exclusion. It may make people well‐accepted in a community as they can be perceived as protective. Further, cumulative lifetime adversity has in some cases been found to promote resilience and have positive effects on mental health and well‐being (Seery et al., 2010). Therefore, interventions geared toward managing and translating “good” anxiety into goal‐directed behavior may help trauma‐exposed individuals in the contexts of education and employment.

Unsurprisingly, considering the established body of evidence indicating loneliness as a risk factor for socioeconomic deprivation and lack of social networks (de Lange et al., 2021; Pengpid & Peltzer, 2021), loneliness was most strongly associated with “limited social participation” in the current analysis. This may currently be considered an even greater concern, further exemplifying the widespread consequences of mandated social isolation throughout the recent COVID‐19 global pandemic (Groarke et al., 2020). However, the elements comprising “limited social participation” are arguably the most easily modifiable dimensions of social exclusion and could be recommended factors to target via intervention. Unfortunately, there is still little evidence to indicate which type of intervention is effective in reducing loneliness (Dahlberg, 2021); therefore, further work is warranted to understand how we can ameliorate these effects.

Taken together, this paper presents important findings that further support the proposition that early life experiences and environment can translate into adult psychological outcomes and future societal consequences (Fisher & Lees, 2016). People who are disproportionately exposed to disadvantaged social factors experience greater future health and social inequalities, which can in turn become disabling, thereby creating further barriers to social inclusion (World Health Organization, 2011). These findings advocate for trauma‐informed approaches across a variety of sectors, including education and health, and support the call for services to acknowledge childhood trauma in the management of mental health and social integration issues in both individuals and communities (Sweeney et al., 2016).

With regard to the methodology of the paper, the large sample size is an obvious strength, in addition to our innovative approach to social exclusion that has enabled the exploration of the social consequences and moderating pathways of childhood trauma in UK Biobank participants. The study also utilized reliable, valid, and economic questionnaires to assess childhood trauma (CTS‐5), anxiety (GAD‐7), and depression (PHQ‐9) recommended for use in large epidemiological studies (Grabe et al., 2012). However, the CTS‐5 only includes five questions that may disregard other aspects of the adversity suffered in childhood (e.g., lack of food, bullying at school). It may therefore be of interest to include a more comprehensive assessment of childhood adversity in future studies.

Further limitations include the self‐selecting sample that was not entirely representative of the U.K. population. However, this is a well‐known limitation of the UK Biobank in general. The use of listwise deletion may also have contributed to this; however, due to the size of the data set and the low proportion of missing data, this was deemed to have a limited impact on the results. The use of composite scores to assess social exclusion and single dichotomous question to assess loneliness may also be regarded as a limitation, compared to using validated measures, as is the lack of data on familial factors (e.g., composition) and socioeconomic status (SES) in childhood that would likely play a confounding role in the associations explored here. Childhood SES has been found to be a strong predictor of both childhood and later‐life adversity (e.g., Walsh et al., 2019), which in turn could put individuals at a higher risk of mental health problems. However, a recent study found childhood adversity to be associated with lower adult SES, independent of childhood SES (Suglia et al., 2022). Nevertheless, it is likely that the associations between the variables in the present study are bidirectional or circular in nature.

It must also be acknowledged that the questions in the CTS‐5 are retrospective and potentially affected by recall abilities. There may also be a potential bias issue due to the possibility of enhanced recall of traumatic events in individuals suffering from loneliness and depression. However, this is a fundamental issue in research into childhood trauma, which is difficult to overcome (Grabe et al., 2012).

Finally, the analyses in the current paper are exploratory and retrospective, and therefore cannot infer direct causal pathways. While we propose a cumulative pathway model, in which childhood adversity increases the propensity for distress and isolation in adulthood, which exacerbates social exclusion, the reverse relationship may also be true. In fact, the associations between these variables are likely complex and multidirectional. Further studies require prospective analyses to explore social exclusion in relation to childhood trauma and the moderating effects of loneliness and other psychological and social factors.

In summary, the current findings contribute to the body of literature demonstrating links between loneliness, depression, and social exclusion, in individuals exposed to early life trauma. While much research has been carried out into the links between childhood trauma and mental health in the UK Biobank, this is the first to examine the association between childhood trauma and these dimensions of social exclusion as potential outcomes. It is also further suggested that anxiety symptoms may indicate resilience in terms of the pursuit of education and employment in this population. The key recommendations advocate for the adoption of trauma‐informed approaches to services in a variety of settings; however, further prospective research is warranted.

## AUTHOR CONTRIBUTIONS

**Sarah F. Allen**: Conceptualization; data curation; formal analysis; funding acquisition; project administration; writing—original draft; writing—review and editing. **Simon Gilbody**: Conceptualization; funding acquisition; writing—review and editing. **Karl Atkin**: Conceptualization; writing—review and editing. **Christina M. van der Feltz‐Cornelis**: Conceptualization; funding acquisition; supervision; writing—review and editing.

## FUNDING INFORMATION

This study was funded by the University of York Pump Priming Fund.

## CONFLICT OF INTEREST STATEMENT

The authors declare no conflicts of interest.

### PEER REVIEW

The peer review history for this article is available at https://publons.com/publon/10.1002/brb3.2959.

## Data Availability

The data that support the findings of this study are available from UK Biobank. Restrictions apply to the availability of these data, which were used under license for this study. UK Biobank ref no: 49442.
